# Supplementary figures and images for: The genetic basis of variation in immune defense against Lysinibacillus fusiformis infection in Drosophila melanogaster
Source: PLoS Pathog. 2023 Aug 7;19(8):e1010934. doi: 10.1371/journal.ppat.1010934 (PMC10434897; doi:10.1371/journal.ppat.1010934)

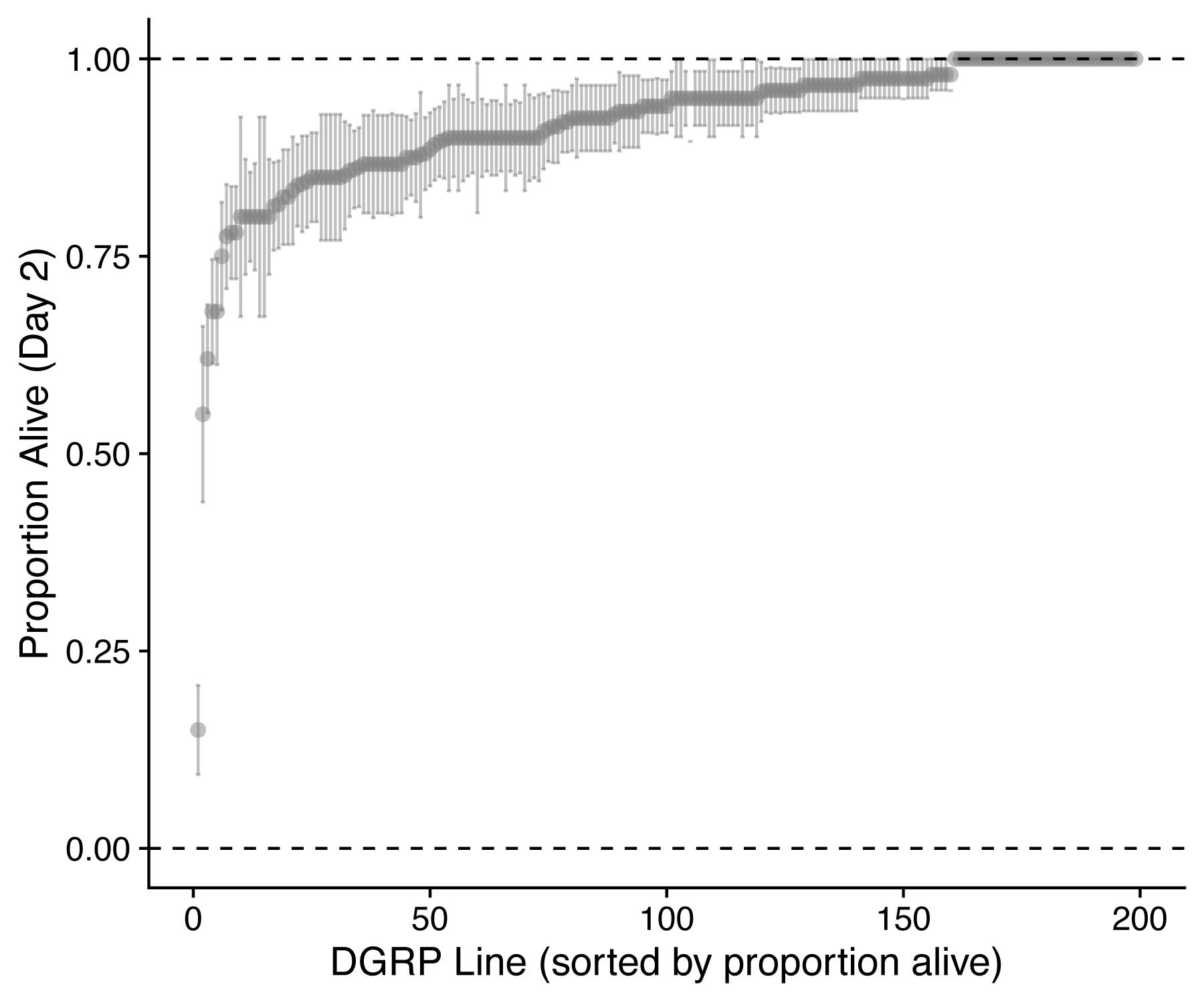

Supplement: S1 Fig — Survival measured as the proportion alive at Day 2 divided by the total number infected per line. (TIF) [file ppat.1010934.s020.tif]

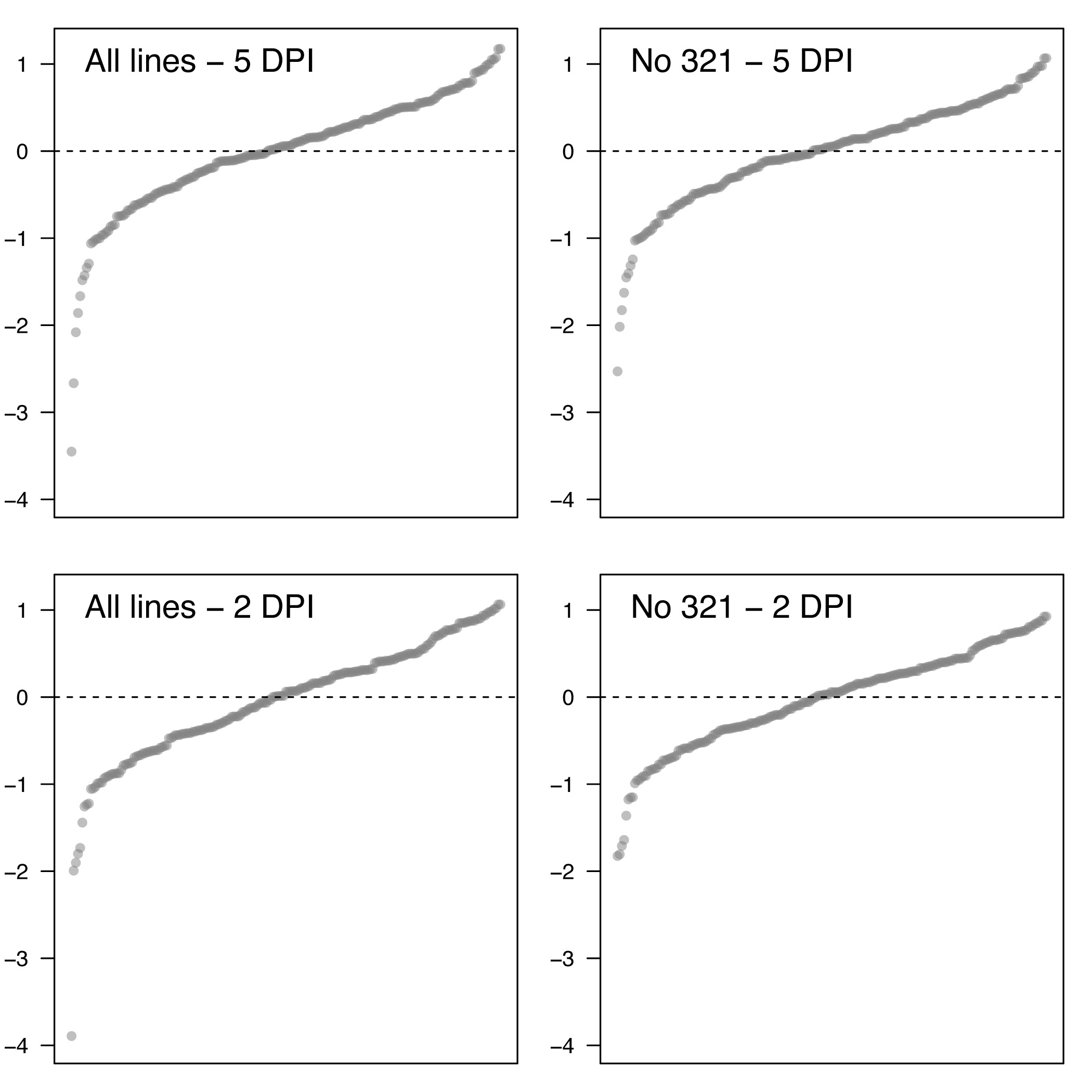

Supplement: S2 Fig — Data is presented with all lines present or excluding line 321 (which had low survival) and at 5- or 2-days post infection. (TIF) [file ppat.1010934.s021.tif]

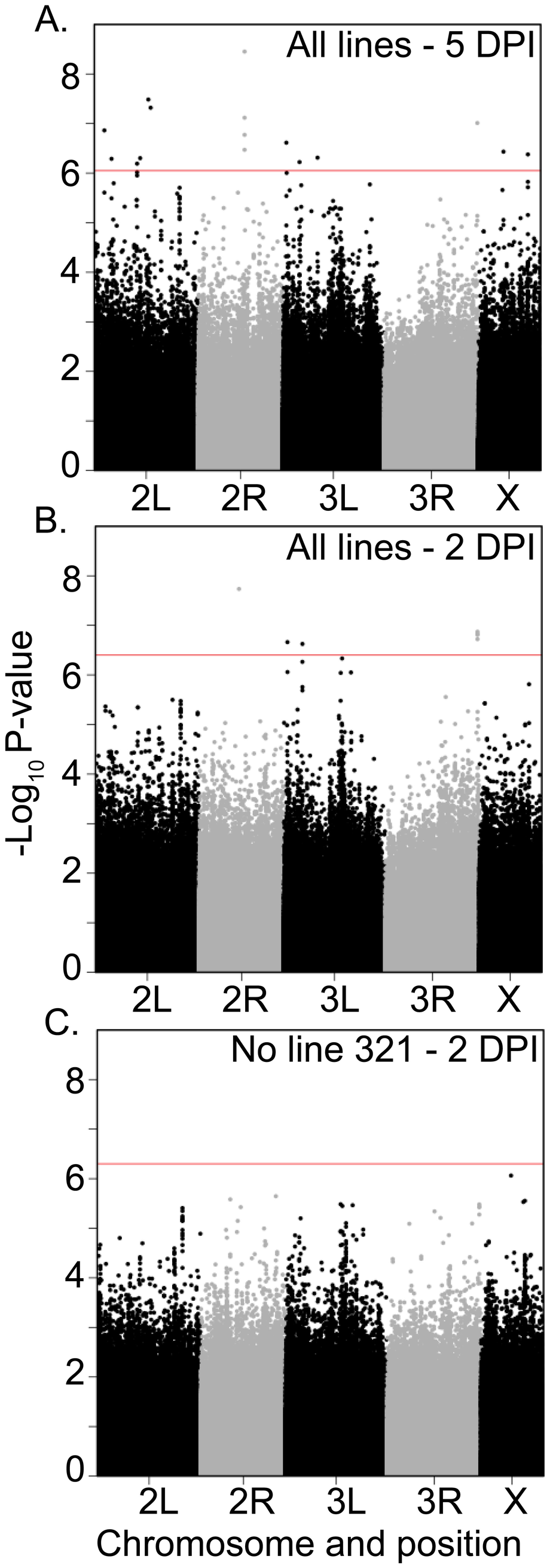

Supplement: S3 Fig — (TIF) [file ppat.1010934.s022.tif]

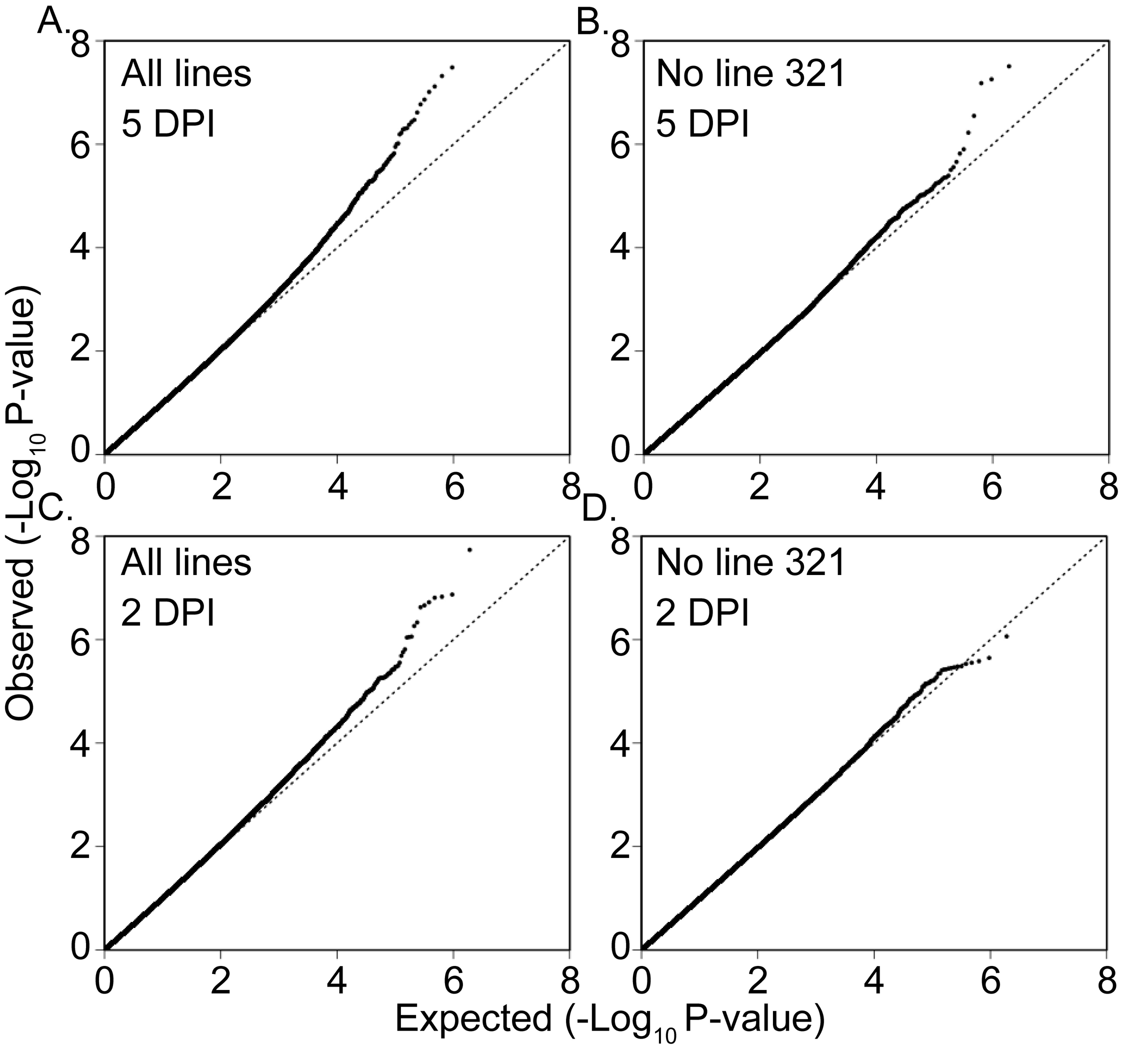

Supplement: S4 Fig — (TIF) [file ppat.1010934.s023.tif]

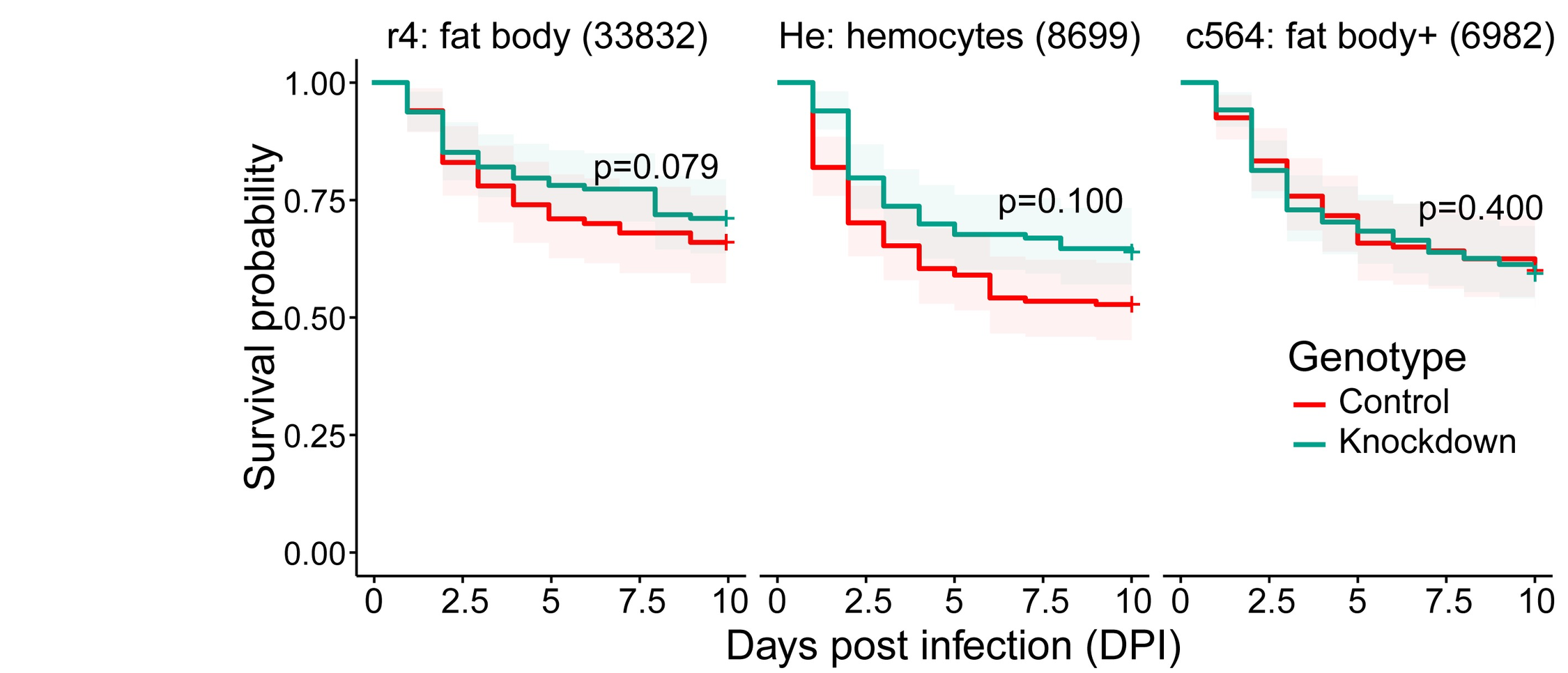

Supplement: S5 Fig — Three different drivers (r4 –fat body specific, He–hemocyte specific, and c564 –expressed in fat body, gut, and several male tissues) were crossed to a TRIP Pvr RNAi line (37520) or empty vector control (36304). Survival was monitored for 10 days post infection. (TIF) [file ppat.1010934.s024.tif]

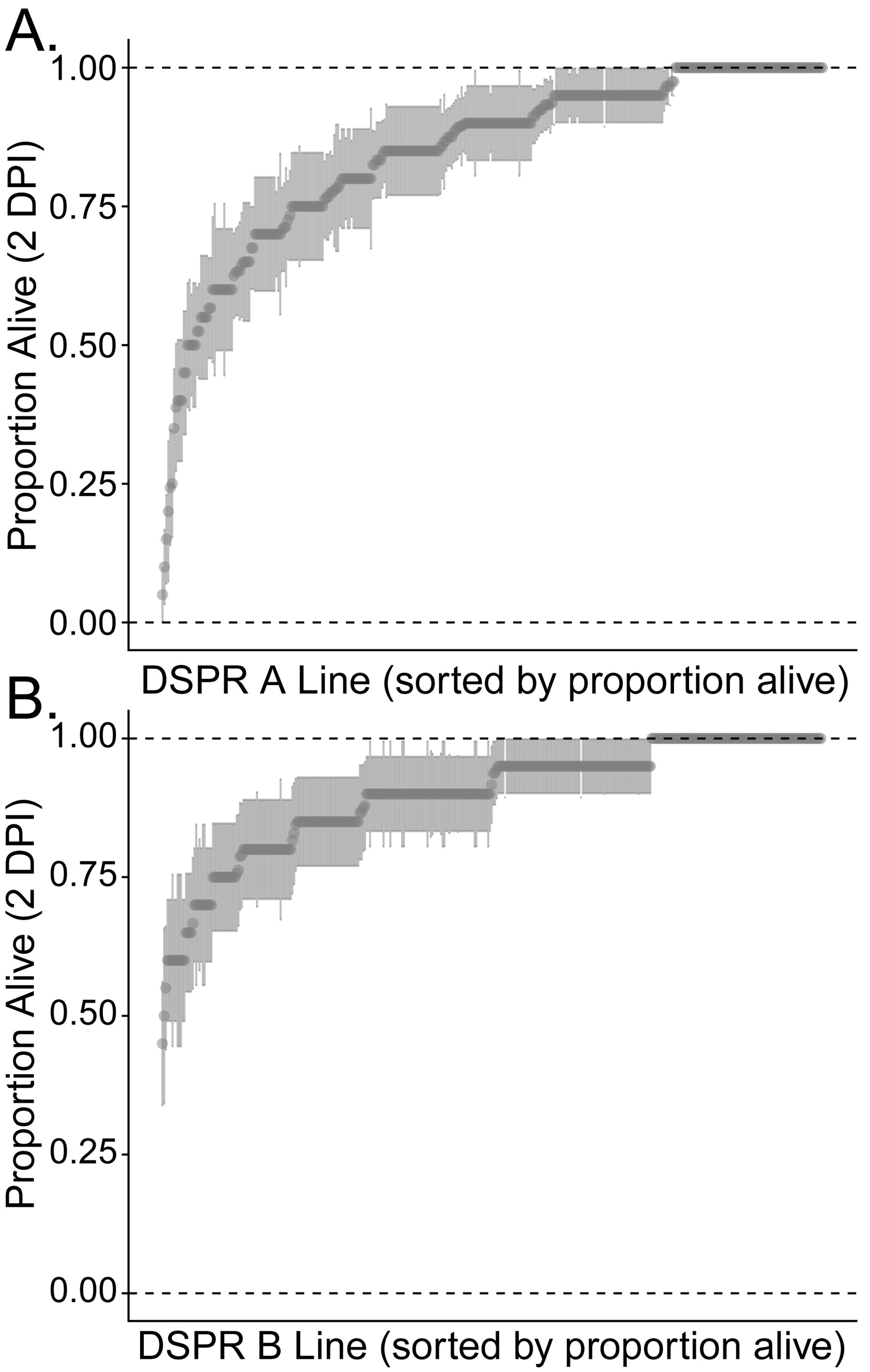

Supplement: S6 Fig — Raw survival is measured as proportion alive at 2 days post infection. A) Panel A, B) Panel B. (TIF) [file ppat.1010934.s025.tif]

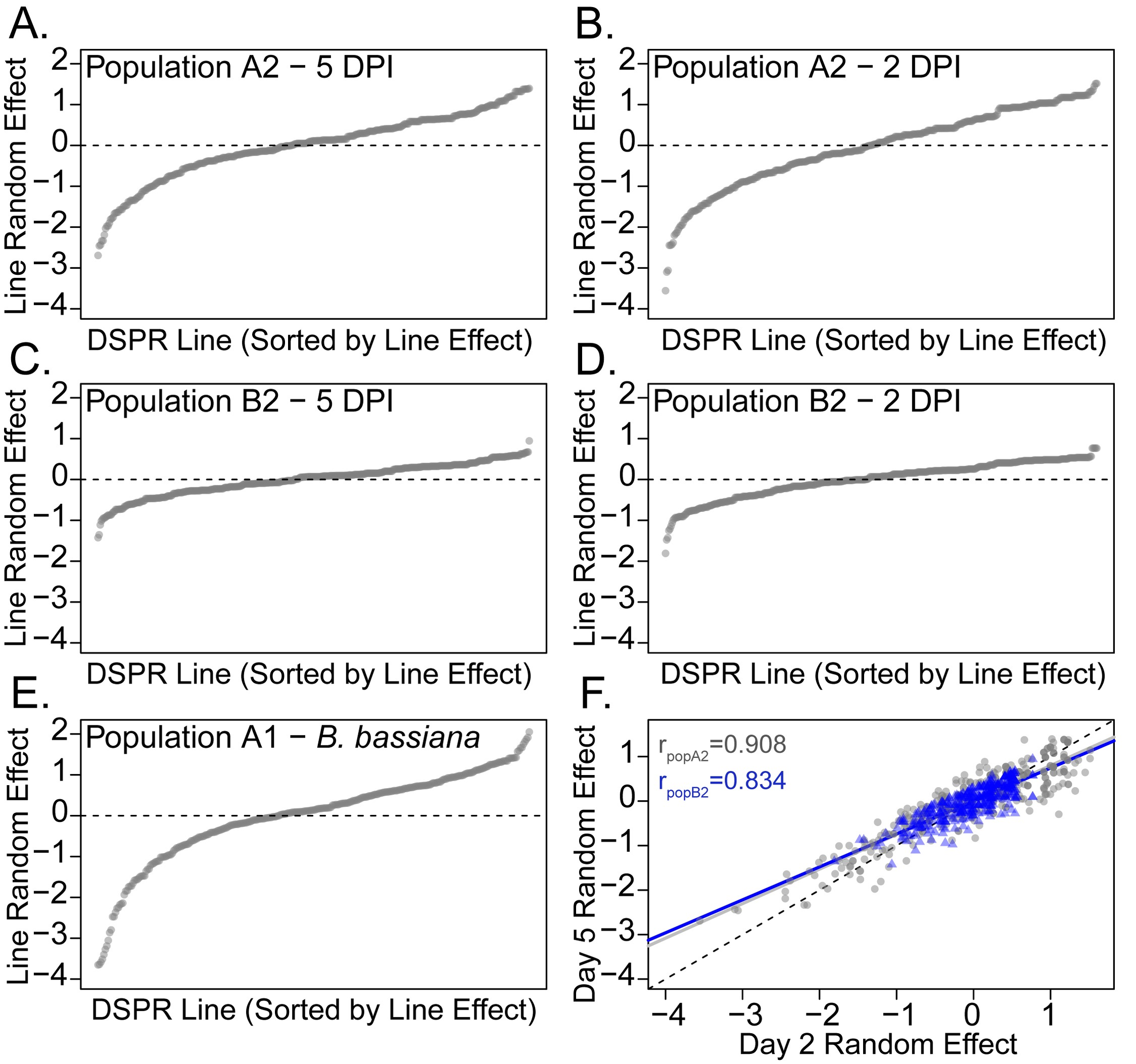

Supplement: S7 Fig — (TIF) [file ppat.1010934.s026.tif]

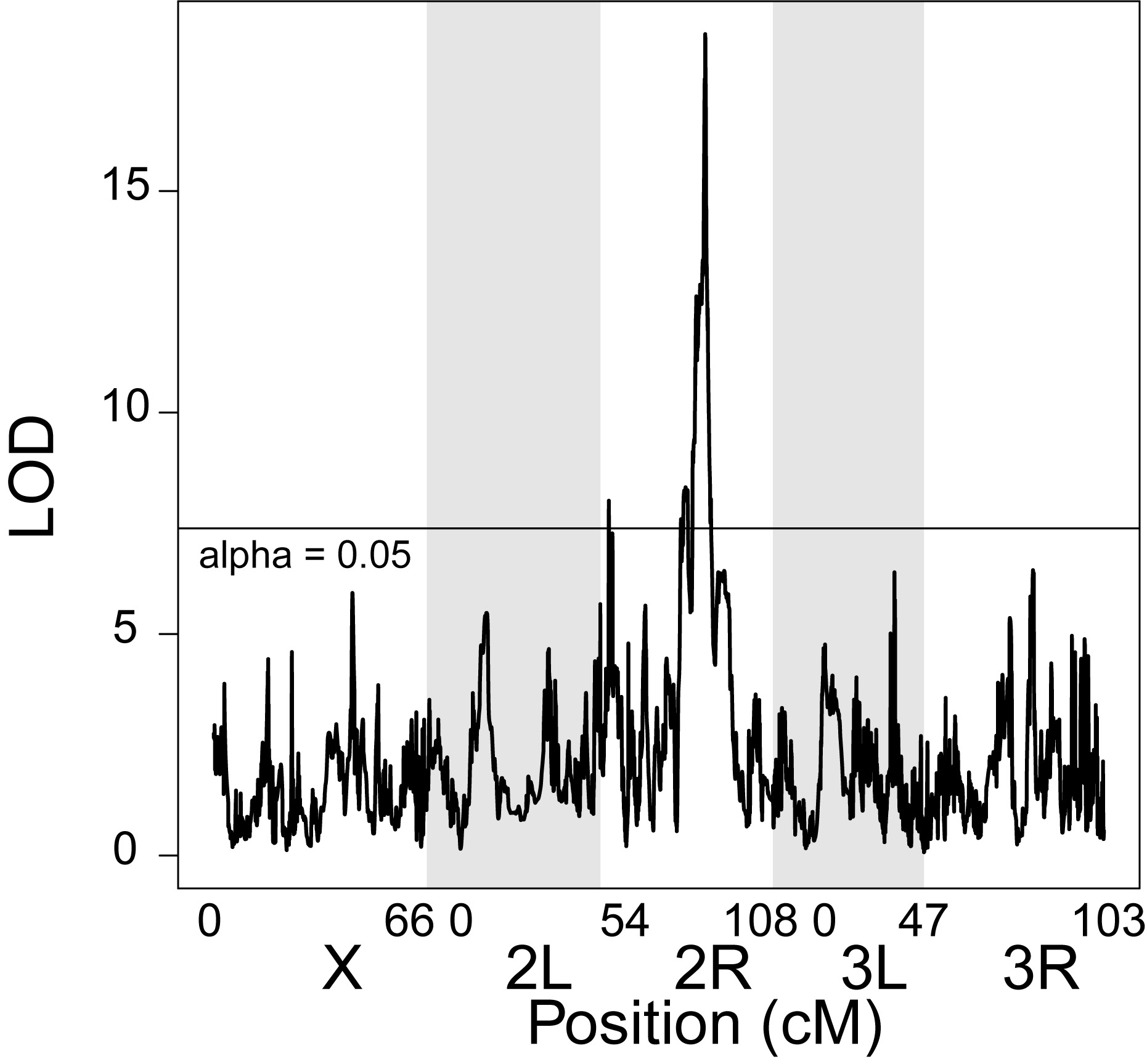

Supplement: S8 Fig — (TIF) [file ppat.1010934.s027.tif]

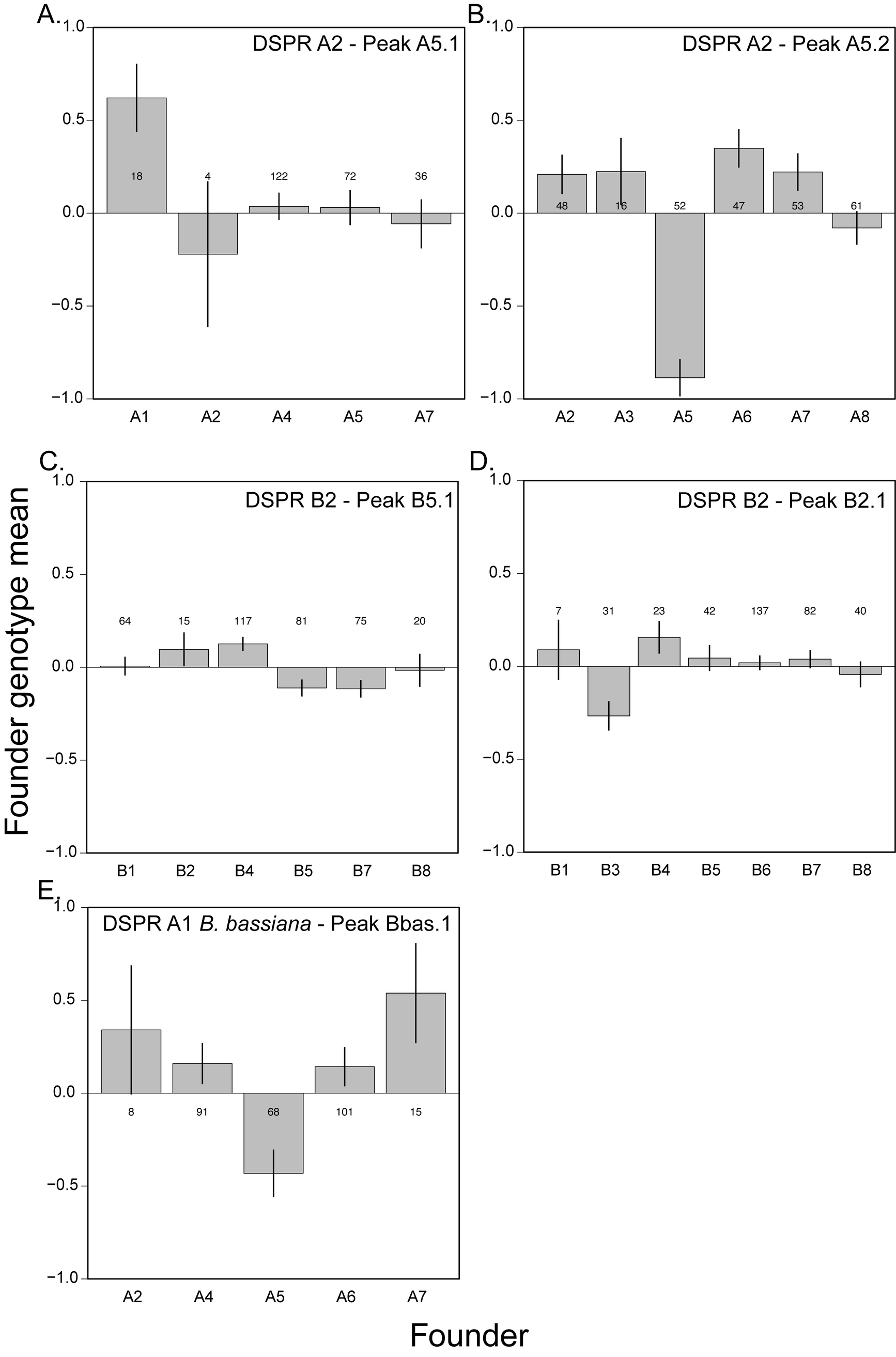

Supplement: S9 Fig — (A and B) Population A2 Day 5 peaks 1 and 2, C) Population B2 Day 5 peak 1, D) Population B2 Day 2 peak 1, E) Population A1 B. bassiana survival peak 1. (TIF) [file ppat.1010934.s028.tif]

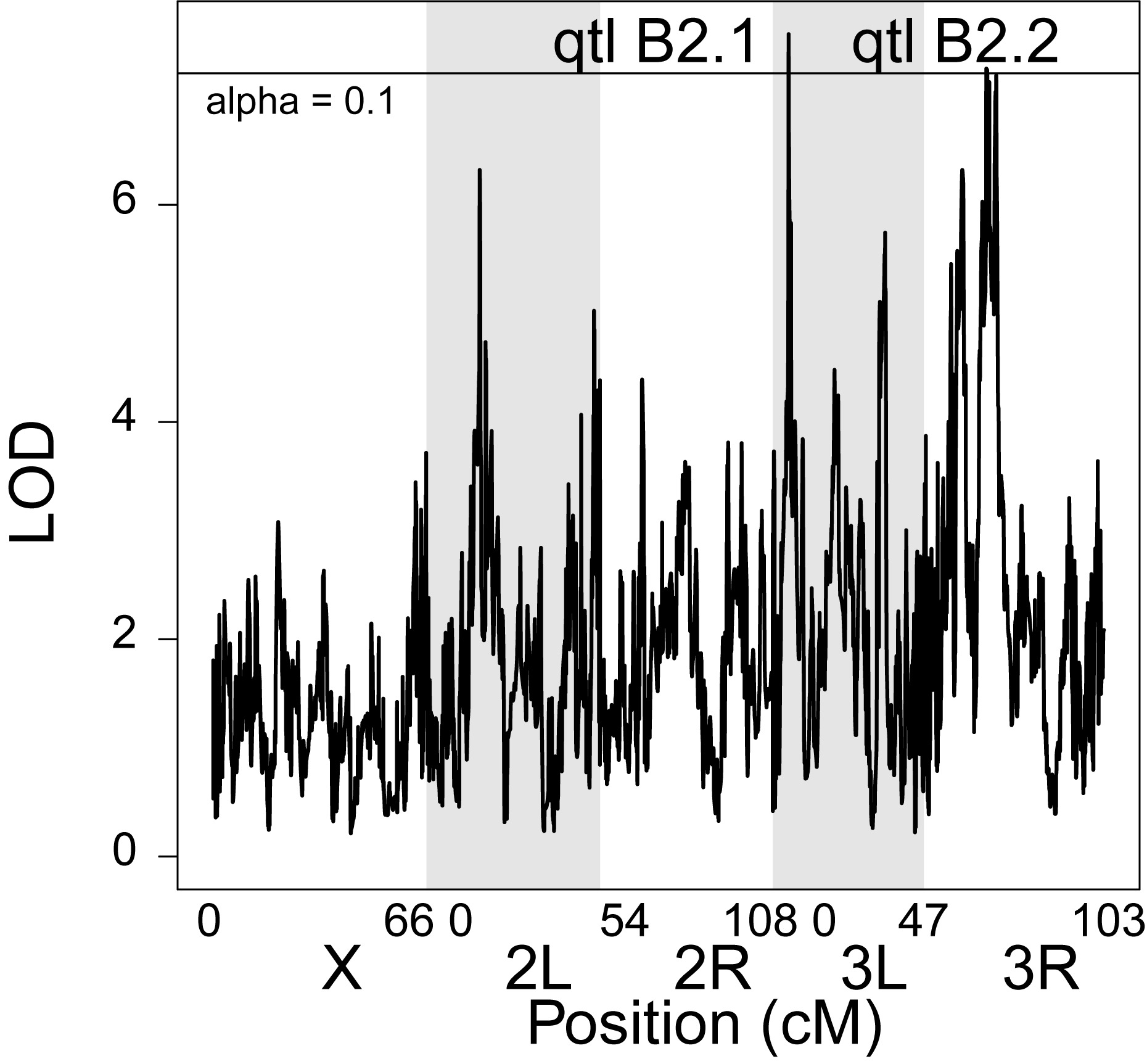

Supplement: S10 Fig — (TIF) [file ppat.1010934.s029.tif]

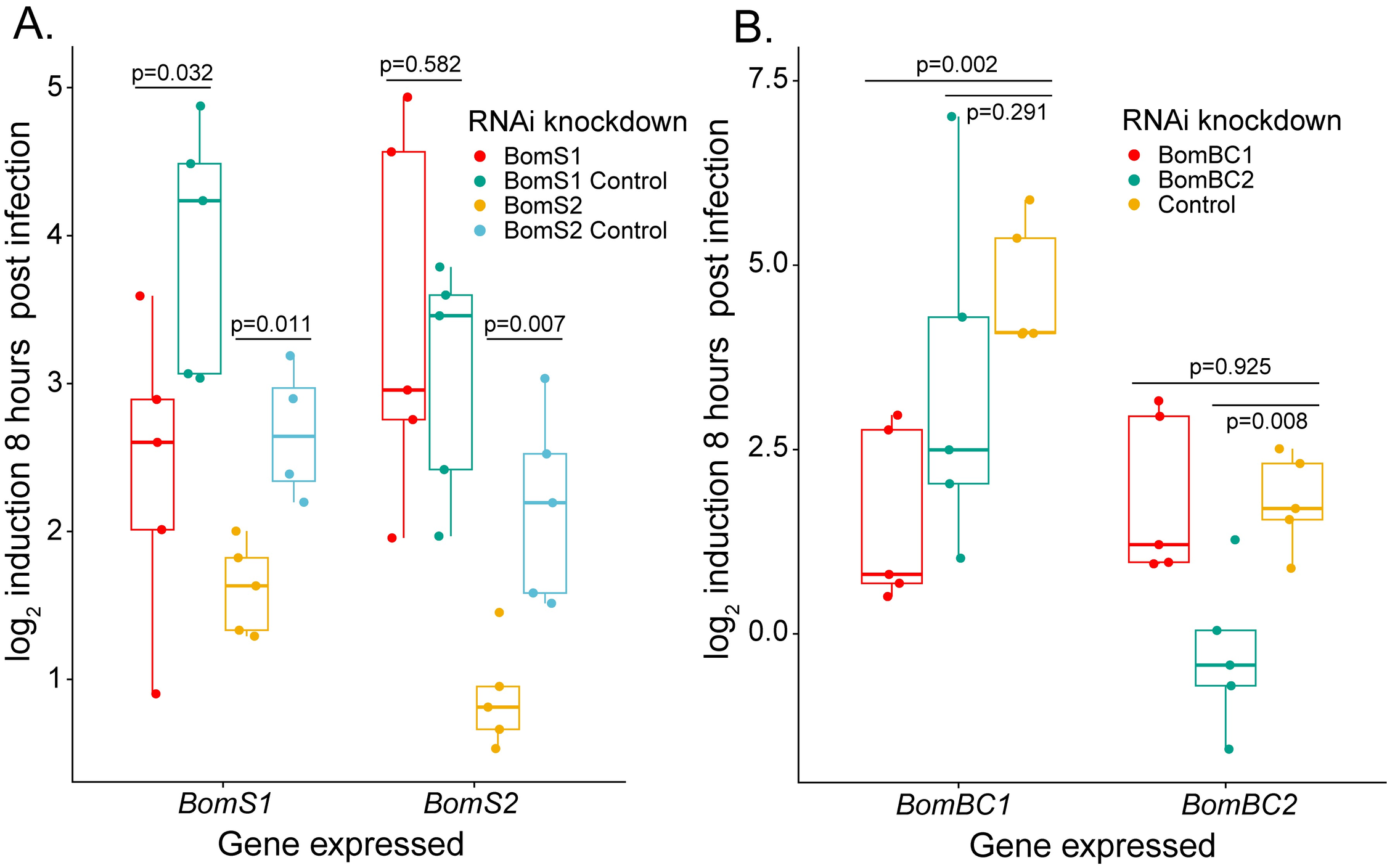

Supplement: S11 Fig — The Y axis depicts the log2 fold induction 8 hours post infection compared to an uninfected (no sterile prick) individual. Relevant comparisons are presented with black bars and p values are from T tests. RNAi knockdown refers to the genes targeted by RNAi constructs and gene expression refers to the primers used to Measure gene expression. (TIF) [file ppat.1010934.s030.tif]
